# Supplementary material for: Non-Cartesian 3D-SPARKLING vs Cartesian 3D-EPI encoding schemes for functional Magnetic Resonance Imaging at 7 Tesla
Source: PLoS One. 2024 May 13;19(5):e0299925. doi: 10.1371/journal.pone.0299925 (PMC11090341; doi:10.1371/journal.pone.0299925)
Supplement: S1 Appendix — (PDF) [file pone.0299925.s001.pdf]

Appendix

Nonlinear CS-based reconstructions can induce bias, and depending on the level of regularization, they may not guarantee the Gaussianity of the noise, which is an essential hypothesis as the GLM analysis is grounded on the Gaussianity of the residuals. In this work, the same regularization parameter (and, therefore, the same reconstruction algorithm) was used for all consecutive volumes. This means that any potential bias caused by nonlinear regularized reconstruction would be constant across all fMRI scans. Such a tendency would be captured by the baseline regressor in the design matrix, and the residual error distribution would remain centered. Additionally, as we used a moderate amount of ℓ_1 regularization, we showcase in the following that this does not strongly affect the Gaussianity hypothesis on the residuals of the GLM.

First, we compared the impact of three different reconstruction strategies on the statistical analysis, namely, the effect (β coefficients) captured by the baseline regressor (Figure 10) and residual errors (Figure 11) of the GLM-fitted 3D-SPARKLING fMRI time series collected in V#3. These three different strategies were chosen in order to disentangle the effect of nonlinear reconstruction due to sparse regularization from that of the regularization itself on top of the 3D SPARKLING encoding scheme:

a: The nonlinear regularized reconstruction using a ℓ_1-norm regularization in the wavelet domain ($\lambda = 10^{-8}$).

b: The linear regularized reconstruction using a squared ℓ_2-norm regularization in the wavelet domain (the regularization parameter $\lambda = 10^{-8}$). The wavelet basis chosen is `Sym8` which is an orthogonal basis, therefore even though the squared ℓ_2-norm is applied to the wavelet coefficients, the reconstruction remains linear.

c: The zero-filled adjoint Fourier reconstruction (no regularization, i.e. $\lambda = 0$).

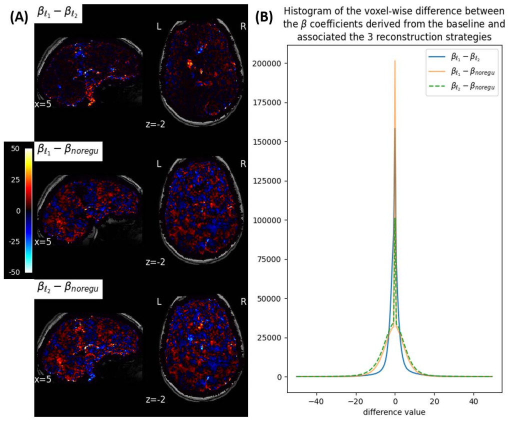

Fig 10. (A): Maps of the voxel-wise difference between the β coefficients derived from the baseline regressor in GLM analysis and associated with 3D-SPARKLING data reconstructed using (a) to (c) in V#3. (B): Histograms of these maps.

Second, and specifically regarding the distributions of the residuals, we compared the results associated with 3D-SPARKLING to those associated with 3D-EPI and corresponding to the same volunteer (V#3).

Figure 10(A) shows the maps of the voxel-wise difference of the β coefficients derived from the baseline regressor in the GLM and associated with 3D-SPARKLING data reconstructed using (a) to (c): After computing the β coefficient maps associated with the baseline regressor and each reconstruction strategy ((a) to (c)), the voxel-wise

differences between these maps were computed, namely, $\beta_{\ell_1} - \beta_{\ell_2}$, $\beta_{\ell_1} - \beta_{noregu}$ and $\beta_{\ell_2} - \beta_{noregu}$. There are visible differences between the 3 maps. As we assume that reconstruction strategy (c) yields no bias as it's not regularized, these results suggest that the bias induced by the nonlinearity ($\beta_{\ell_1} - \beta_{\ell_2}$) is actually lower than that induced by the regularization ($\beta_{\ell_1} - \beta_{noregu}$ or $\beta_{\ell_2} - \beta_{noregu}$). In Figure 10(B), the histograms of these voxel-wise differences in β coefficients are plotted and confirm the above observations: As the distribution of these differences is narrower or tighter between the two regularized (linear versus nonlinear) reconstruction strategies as compared to the differences between the regularized and unregularized strategies, most of the bias is due to the regularization itself and not to its nonlinear aspect.

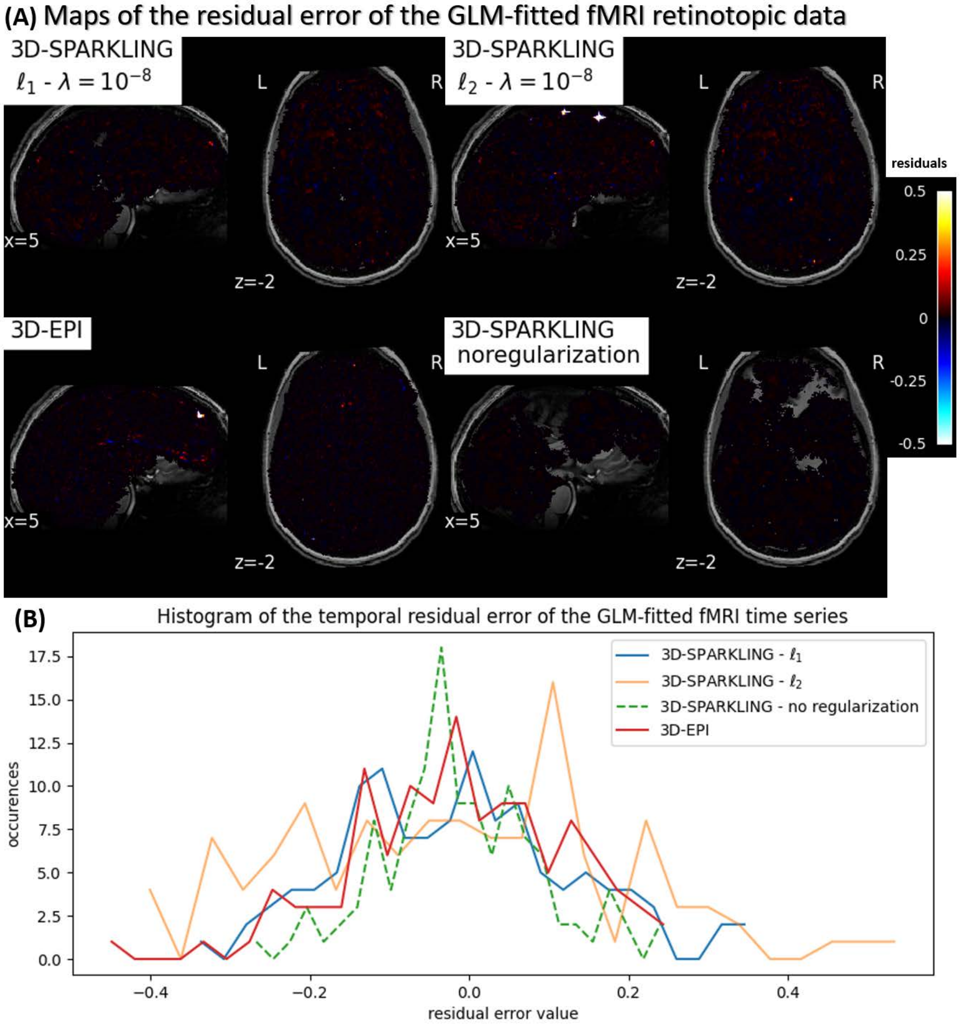

Fig 11. (A) Maps and (B) temporal histograms of the residual error of the GLM-fitted retinotopic fMRI data associated with 3D-SPARKLING (reconstructed with strategies (a)-(c)) and 3D-EPI and collected in V#3.

Figure 11(A) shows the residual error maps associated with 3D-SPARKLING (reconstructed using (a)-(c) strategies) and 3D-EPI fMRI volumes. These maps were produced by averaging the temporal residual errors over the time dimension to obtain a global summary. The residual error seems centered around zero for the four datasets. Additionally, reconstruction strategy (c) results in more lost signal than (a) and (b) as the residuals reach higher values. Figure 11(B) shows the histograms of the temporal residual error of the GLM-fitted fMRI volumes: Firstly, we spatially averaged the residual errors over the brain mask, then computed the temporal histograms[1]. The results associated with the four scenarios, namely the data acquired with 3D-SPARKLING and reconstructed using strategies (a)-(c) and those acquired with 3D-EPI, are reasonably similar. Despite small differences between the histograms associated with the reconstruction strategies (a)-(c), the distributions are centered around zero. Furthermore, the histograms associated with 3D-SPARKLING and reconstruction strategy (a) and that associated with 3D-EPI are quite similar and are spread alike around zero. Additionally, to obtain an objective measure of the similarity between these histograms and evaluate how tenable the hypothesis of the Gaussianity of the residuals is, a Kolmogorov-Smirnov (KS) test was performed between:

(i) The histograms associated with 3D-SPARKLING data and reconstruction strategies (a) and (b), respectively.

(ii) The histograms associated with 3D-SPARKLING data and reconstruction strategies (a) and (c), respectively.

(iii) The histograms associated with 3D-SPARKLING data and reconstructed using (a) and those associated with 3D-EPI.

The null hypothesis (H_0) used is that the two distributions are identical and the p-values were, 0.18, 0.1, and 0.9 for (i), (ii), and (iii), respectively. This means that H_0 cannot be rejected and therefore that the distributions are significantly similar. We conclude that the hypothesis of Gaussianity remains tenable for 3D-SPARKLING data reconstructed with a CS-based reconstruction. This could be explained by the fact that the level of regularization performed was set to a reasonably good but low value.

[1]The number of samples in the histograms is given by the number of scans.
