# Supplementary material for: Non-Cartesian 3D-SPARKLING vs Cartesian 3D-EPI encoding schemes for functional Magnetic Resonance Imaging at 7 Tesla
Source: PLoS One. 2024 May 13;19(5):e0299925. doi: 10.1371/journal.pone.0299925 (PMC11090341; doi:10.1371/journal.pone.0299925)
Supplement: Attachment — Submitted filename: Response_to_Reviewers.pdf [file pone.0299925.s002.pdf]

Responses to reviews for PONE-D-23-03044

Non-Cartesian 3D-SPARKLING vs Cartesian 3D-EPI encoding schemes for functional Magnetic Resonance Imaging at 7 Tesla

Zaineb Amor Philippe Ciuciu Chaithya G R Guillaume Daval-Frérot
Franck Mauconduit Bertrand Thirion Alexandre Vignaud

August 4, 2023

We thank the editor and reviewers for their consideration and comments. In the following, we will answer to each point raised separately.

1 Editors comments

General Comments:

We have received two expert reviews. However, both reviewers raise many critical issues. After carefully reading this work, I have to agree with the reviewers that the significance of this work is marginal. So, I recommendation a major revision of the your manuscript.

Authors: We thank the editor for the feedback. We modified the manuscript according to the journal requirements:

- We have modified the formatting of our manuscript to meet PLOS ONE's requirements, notably regarding the formatting of the different sections and subsections titles.
- We have modified the Data Availability Statement section with more details about the code and data sharing.
- We have modified the Funding section according to the editor's comments.
- We have added "written" in our ethics statement in the "Methods" section to indicate that the volunteer's consent was written. The current ethics statement is our full ethics statement.
- We removed Figure 2 and replaced it with the Figure of the sampling scheme, which was requested by reviewer#2.

2 Responses to reviewer #1

General Comments: The paper is generally well written and structured but I suggest minor re-. The literature review was thorough, It is comprehensive and completely justifies No technical errors and approved for publication

Authors: We thank the reviewer for their positive feedback. Our understanding is that the reviewer approves the publication without further questions.

3 Responses to reviewer #2

General Comments: In this paper, a 3D-SPARKLING trajectory is evaluated for use in whole-brain isotropic functional MRI at 7 T. The proposed method uses a non-Cartesian readout, coupled with a non-linear reconstruction (compressed sensing with wavelet domain sparsity), and was compared to conventional 3D-EPI with matched parameters. While image quality in the 3D-SPARKLING acquisition was poorer than the 3D-EPI, most metrics showed improved sensitivity and specificty for the proposed method over 3D EPI, evaluated in 6 healthy volunteers.

The paper is reasonably well written, and many comprehensive experiments/evaluations are performed. However, I have several reservations about the results presented in the paper:

R2.1: The paper doesn't provide a figure or diagram of the resulting 3D SPARKLING trajectory. However, my understanding is that 3D SPARKLING optimized trajectories depend heavily on initialization, and in previous work, 3D SPARKLING trajectories were initialized with perturbed 3D radial trajectories. One would assume this to be the case here as well (although it is unstated as far as I can tell). Given this, it is quite confusing that the 3D-SPARKLING trajectories is stated to have been acquired in the "P-A encoding direction" (see Line 163). This is also stated in the bottom row of Table 1. It would be useful for the authors to explain in what way exactly the "P-A encoding direction" is meaningful in the context of 3D SPARKLING.

Authors: We thank the reviewer for pointing out this aspect that can confuse the reader. Our trajectories are also initialized using In-Out perturbed radial trajectories. Therefore, each readout of the 3D-SPARKLING sampling pattern is defined as an In-Out trajectory spanning the k-space from one extremity to the other by passing through the center of the k-space at TE. The P-A encoding direction indicates to the scanner one of the spatial orientations of the k-space. We agree that such an indication can be confusing and somehow irrelevant in the case of 3D-SPARKLING. This is why we decided to remove the P-A encoding direction from Table 1 in the manuscript for 3D-SPARKLING and to explain that the orientations are maintained similarly for 3D-EPI and 3D-SPARKLING in its legend. Additionally, We have introduced a figure showing the sampling scheme as suggested (Figure 1 in this letter corresponding to Figure 2 in the core manuscript).

R2.2: It is somewhat difficult to believe that despite poorer image quality, 3D-SPARKLING outperforms 3D-EPI in nearly every quantitative metric. As the BOLD signal is encoded in

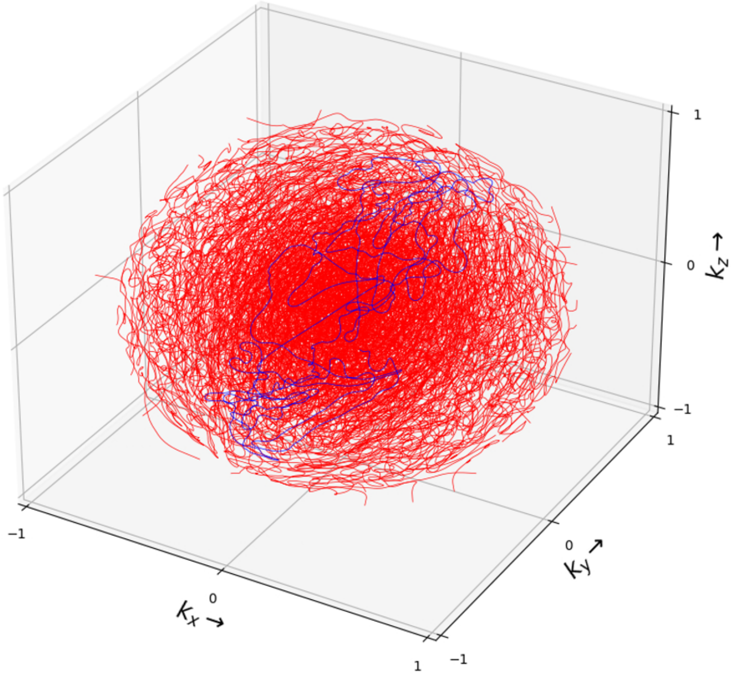

Figure 1: **3D-SPARKLING sampling scheme.** The sampling scheme is segmented into 48 readouts plotted in red except for one plotted in blue for the sake of visualization.

the image magnitude, one might expect that the blurring/aliasing seen in the image should also be reflected in the BOLD signals. The authors address this point in part by appealing to BOLD PSFs, but if it is expected that inclusion of dynamic off-resonance correction would significantly improve image quality, I believe the manuscript would significantly benefit from including that, rather than leaving it for future work. It would be difficult to recommend a method that produces qualitatively worse looking images, despite what the quantitative metrics show.

Authors: We thank the reviewer for this relevant suggestion. We have already performed this work on a separate dataset from the one we used for this paper and prepared it for publication elsewhere. It is not possible to perform the same correction on the current dataset we used for this paper because we did not collect the measurements corresponding to dynamic B_0 fluctuations, which are needed to perform such a correction: At the time we collected this set of fMRI data, we haven't managed to put in place an experimental setup (using the Skope Clip-on Camera) that enables us to monitor dynamic B_0 fluctuations in a way that is compatible with an fMRI experiment (there is a huge technical challenge here related to the synchronization of dynamic off-resonance information with fMRI data that we solved and deserves a full and separate publication). When we managed to put in place such an experimental setup, and given its complexity and time constraints, we only implemented it for 3D-SPARKLING. We have already presented some of the results where we show the improvement for 1mm^3 and 2.4s-TR 3D-SPARKLING retinotopic fMRI data in terms of image quality, tSNR and sensitivity to the BOLD contrast at the 2023 ISMRM annual conference [1]. We prefer to keep these two papers distinct as we do not

use the same dataset and combining them would make this paper extremely long and hard to digest. However, as the ISMRM abstract is now published, we updated the reference in the Discussion section of this paper to refer to the new state of the art (reference [87] in the core manuscript).

R2.3: Another reasons CS has not been widely adopted in fMRI is due to the nonlinear reconstruction. In contrast with most linear reconstructions (e.g. parallel imaging), CS reconstructions are not unbiased, and furthermore, due to the non-linearity of the reconstruction, characteristics of the resulting "noise" is not straightforward. As most fMRI analysis tools have assumptions grounded in the Gaussianity of residuals, while this can be true for linear reconstructions (in the high SNR limit anyway when dealing with magnitude images), no guarantees about the distributional properties of the signal residuals can be made with CS reconstructions. This may be confounded further by interaction with image pre-processing. Therefore, naive adoption of fMRI analysis methods without consideration of altered noise characteristics is statistically unsound. At a minimum, some validation of the residual characteristics of the fMRI analysis models would help establish their statistical validity. Discussion of this important point should also be included in the manuscript. Although the reconstructions performed here are done independently volume-to-volume, if time-dependent regularization is performed, then additional considerations of any potential loss of temporal degrees of freedom are also necessary to ensure statistical validity of the inference process.
Authors: We thank the reviewer for raising this critical point about how tenable the hypothesis on the Gaussianity of the residuals in the GLM is for CS-based nonlinear image reconstruction. Indeed, the use of non-Cartesian CS-based fMRI is scarce. Nevertheless, some papers investigated the matter: In [2], variable density sampling spiral readouts were used and combined with a low rank+sparse reconstruction. Such a reconstruction induces a temporal dependency between the consecutive shots and complicates the statistical analysis: We refrained from performing such a reconstruction and performed a simple volume-wise independent CS-based reconstruction. However depending on the regularization level, a CS-based reconstruction, even when it is not temporally-regularized may not guarantee the Gaussianity of the residuals in the statistical analysis. To address this point, we added an Appendix, pages 25 to 27 in the manuscript, demonstrating that this assumption remains tenable in the context of the volume-wise CS nonlinear reconstruction we used in this work. More precisely, starting from the same 3D-SPARKLING raw k-space data collected during the retinotopic fMRI experiment, we compared the typical CS-based image reconstruction used so far in the manuscript (denoted (a)) with a linear regularized reconstruction by replacing the ℓ_1-norm with a squared ℓ_2-norm (denoted (b)) and a zero-filled linear reconstruction (unregularized, denoted (c)). The three different reconstruction strategies associated with 3D-SPARKLING data were performed to disentangle the impact of the nonlinearity reconstruction from that of the linear one, whether it's regularized or not. This study, therefore, allowed us to study both the potential bias due to regularization and the deviation from Gaussianity due to ℓ_1-norm regularization. We also assessed these three differently reconstructed datasets with respect to the fMRI volumes acquired with 3D-EPI. The comparison was based on the corresponding β coefficients associated with the base-

line regressor and distributions of the residuals[1] in the GLM, namely the voxelwise errors computed along the time axis. For the sake of reproducibility, we averaged the histograms across voxels within the brain mask. Then, the similarity between these histograms was tested using a Kolmogorov-Smirnov (KS) test was performed between:

(i) The histograms associated with 3D-SPARKLING and reconstruction strategies (a) and (b), respectively

(ii) The histograms associated with 3D-SPARKLING and reconstruction strategies (a) and (c), respectively

(iii) The histogram associated with 3D-SPARKLING and reconstructed using (a) and that associated with 3D-EPI

As the null hypothesis (H_0) used is that the two distributions are identical and the p-values were, 0.18, 0.1 and 0.9 for respectively (i), (ii) and (iii), we can conclude that the distributions are significantly similar. Therefore, the Gaussianity hypothesis of the distribution of the residuals remains as realistic in the case of our nonlinear CS-based reconstruction as it is in the case of 3D-EPI. In fact, since we use the same regularization parameter for all the consecutive volumes, any potential bias induced by the reconstruction will be the same for all the volumes, and, therefore, it would be constant over the time series. The baseline regressor in the GLM design matrix will, then, capture it. In the end, the residuals follow a zero-mean Gaussian distribution.

R2.4: As a follow-up to point 3, evaluation of tSNR is not particularly meaningful when comparing biased (CS) vs. unbiased (PI) reconstructions. Between unbiased reconstructions, tSNR evaluation is appropriate as it is related to mean squared error (no bias). However, heavily regularized CS reconstructions could potentially have infinite tSNR, as variance is reduced to zero (as tSNR does not penalize bias). Therefore, some consideration of this fact should be taken into account when reporting tSNR.
Authors: We thank the reviewer for raising this concern. We agree that the level of regularization of the CS-based reconstruction will impact the tSNR. We, now, acknowledge this point when reporting the tSNR results in page 11 in the manuscript by stating that a direct comparison between the tSNR yielded by 3D-SPARKLING and 3D-EPI is limited. However, as we chose to perform a moderate amount of ℓ_1 regularization, the impact should not be very significant. The impact of the encoding scheme on the increased tSNR is more significant than that of the reconstruction. Hereafter, we demonstrate our claim.

We compute the tSNR maps using the fMRI (magnitude) volumes for both 3D-SPARKLING and 3D-EPI as the voxel-wise ratio $\frac{\bar{\mu}(\nu)}{\sigma(\nu_d)}$ where $\bar{\mu}(\nu)$ and $\sigma(\nu_d)$ are, respectively, the temporal mean and standard deviation of the intensities of the voxel ν and the same voxel but temporally detrended (ν_d) to eliminate any drift from the scanner. Figure 2 displays respectively the histograms of the voxel-wise temporal mean ($\bar{\mu}(\nu)$), standard variation ($\sigma(\nu_d)$) and tSNR ($\frac{\bar{\mu}(\nu)}{\sigma(\nu_d)}$) derived from the same 3D-SPARKLING fMRI sequence of volumes reconstructed using the CS-based reconstruction used so far in the manuscript (ℓ_1 regularization) and using a zero-filled non-uniform Fourier adjoint operator (no regularization) and that

[1]The number of samples in the histograms is given by the number of scans.

acquired using 3D-EPI for the same volunteer (V#3) after rescaling to maintain the same mean values and dynamic across the three datasets as shown in Figure 2(A). Figure 2(B)-(C) show on one hand that the data collected with 3D-EPI is associated with the highest variance and lowest tSNR. On the other hand, the data collected with 3D-SPARKLING and reconstructed without regularization yields the lowest values of standard deviations and the highest tSNR values. Performing the CS-based reconstruction yields a higher variance in comparison and, as a result, a lower tSNR. The CS-based reconstruction yields a better quality image than the unregularized reconstruction at the expense of a reduced tSNR. This suggests that the impact of the encoding scheme (3D-SPARKLING) on the tSNR increase is more significant than that of the nonlinearity of the reconstruction, even though the latter is not zero.

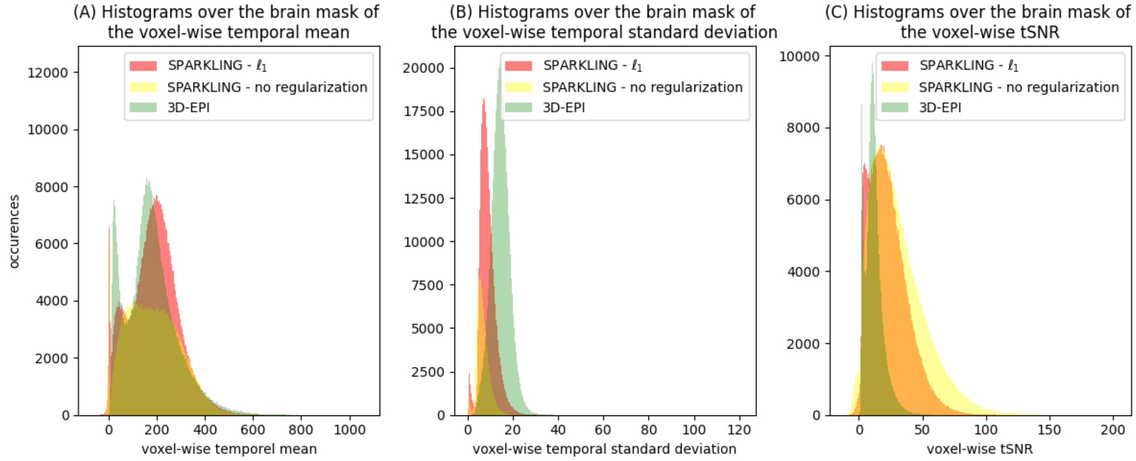

Figure 2: **Histograms of the voxel-wise (A) temporal mean ($\bar{\mu}(\nu)$), (B) standard variation ($\sigma(\nu_d)$) and (C) tSNR ($\frac{\bar{\mu}(\nu)}{\sigma(\nu_d)}$) derived from the same 3D-SPARKLING fMRI sequence of volumes reconstructed using the CS-based reconstruction used so far in the manuscript (ℓ_1 regularization) and using a zero-filled non-uniform Fourier adjoint operator (no regularization) and that acquired using 3D-EPI for the same volunteer (V#3).**

References

[1] Amor Z, Le Ster C, GR C, Daval-Frérot G, Bertrand T, Boulant N, et al. Impact of B0 imperfections correction on BOLD sensitivity in 3D-SPARKING fMRI data. In: Proc. Intl. Soc. Mag. Reson. Med.. vol. 31; 2023

[2] Petrov AY, Herbst M, Andrew Stenger V. Improving temporal resolution in fMRI using a 3D spiral acquisition and low rank plus sparse (L+S) reconstruction. In: Neuroimage. 2017;157:660-674
