# Supplementary material for: Non-Cartesian 3D-SPARKLING vs Cartesian 3D-EPI encoding schemes for functional Magnetic Resonance Imaging at 7 Tesla
Source: PLoS One. 2024 May 13;19(5):e0299925. doi: 10.1371/journal.pone.0299925 (PMC11090341; doi:10.1371/journal.pone.0299925)
Supplement: Attachment — Submitted filename: Response_to_Reviewers.pdf [file pone.0299925.s003.pdf]

Responses to reviews for PONE-D-23-03044

Non-Cartesian 3D-SPARKLING vs Cartesian 3D-EPI encoding schemes for functional Magnetic Resonance Imaging at 7 Tesla

Zaineb Amor   Philippe Ciuciu   Chaithya G R   Guillaume Daval-Frérot
Franck Mauconduit   Bertrand Thirion   Alexandre Vignaud

October 5, 2023

We thank the editor and reviewers for their consideration and comments. In the following, we will answer to each point raised separately.

1 Editors comments

General Comments: Authors address all the queries resaid by reviewers. As suggested by the reviewer, authors are requested to recheck and correct the typographical or grammatical mistakes if any.
Authors: We thank the editor for the feedback. We modified the manuscript accordingly:

- We have corrected all the grammatical and typographical errors we could find and rephrased some parts of the text for clarity.
- We fixed the reference list issue: It occurred without our notice when converting our files. The reference list is now consistent with the first version we sent. All references in the bibliography are now cited in the manuscript text. We simply changed reference [87] as part of the response to reviewer#2 (R2.2 in the last response to the reviews document).
  - **Old reference:** "Amor Z, GR C, Le Ster C, Daval-Frérot G, Boulant N, Mauconduit F, et al. B0 field distortions monitoring and correction for 3D non-Cartesian fMRI acquisitions using a field camera: Application to 3D-SPARKLING at 7T. In: Proc. Intl. Soc. Mag. Reson. Med.. vol. 30; 2022."
  - **New reference:** "Amor, Z., Le Ster, C., GR , C., Daval-Frérot, G., Bertrand, T., Boulant, N., Mauconduit, F., Mirkes, C., Ciuciu, P. & Vignaud, A. Impact of B imperfections correction on BOLD sensitivity in 3D-SPARKING fMRI data. Proc. Intl. Soc. Mag. Reson. Med.. 31 (2023)"

2 Responses to reviewer #1

General Comments: I require authors to make some minor revisions article must be clear and correct, some typographical errors must be corrected. Also some grammatical errors should be corrected.
Authors: We thank the reviewer for their feedback. We corrected all the grammatical and typographical errors we could find and rephrased some parts of the text for clarity. The manuscript was proofread by a native English speaker.

3 Responses to reviewer #2

General Comments:

R2.1: As we understand, there are no comments from reviewer#2.
